# Aqua­bis(2-chloro­acetato-κ*O*)(1,10-phenanthroline-κ^2^ *N*,*N*′)copper(II)

**DOI:** 10.1107/S1600536808000044

**Published:** 2008-01-16

**Authors:** Rongdong Yang, Yanfei Li, Junshan Sun, Jikun Li, Changqing Chu

**Affiliations:** a Department of Materials Science, and Chemical Engineering, Taishan University, 271021 Taian, Shandong, People’s Republic of China

## Abstract

In the title complex, [Cu(C_2_H_2_ClO_2_)_2_(C_12_H_8_N_2_)(H_2_O)], the Cu^II^ ion is five-coordinated by two N atoms [Cu—N = 2.005 (2) and 2.029 (2) Å] from the 1,10-phenanthroline ligand, two O atoms [Cu—O = 1.943 (2)–1.966 (2) Å] from two 2-chloro­acetate ligands and one water mol­ecule [Cu—O = 2.253 (2) Å] in a distorted square-pyramidal geometry. The crystal structure exhibits inter­molecular O—H⋯O hydrogen bonds, short Cl⋯Cl contacts [3.334 (1) Å] and π–π inter­actions [centroid–centroid distance 3.621 (11) Å].

## Related literature

For related crystal structures, see: Sieroń (2007 ▶); Czylkowska *et al.* (2004 ▶); Chen *et al.* (1996 ▶); Overgaard *et al.* (2003 ▶).
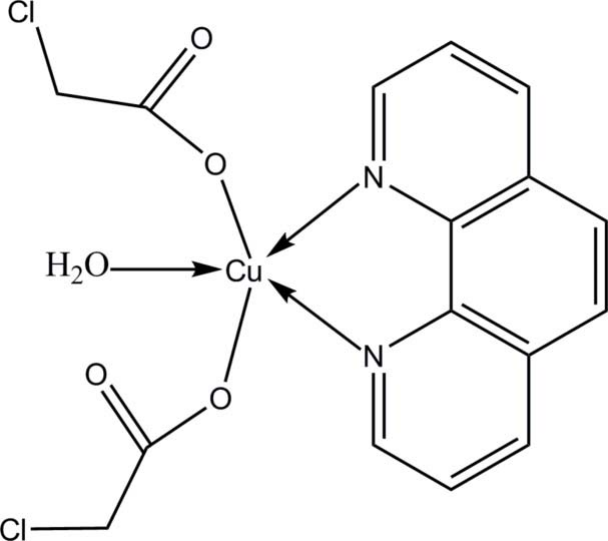


## Experimental

### 

#### Crystal data

[Cu(C_2_H_2_ClO_2_)_2_(C_12_H_8_N_2_)(H_2_O)]
*M* *_r_* = 448.73
Triclinic, 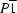

*a* = 8.7730 (6) Å
*b* = 9.2382 (7) Å
*c* = 11.4492 (8) Å
α = 96.2180 (10)°
β = 106.6760 (10)°
γ = 97.9190 (10)°
*V* = 869.66 (11) Å^3^
*Z* = 2
Mo *K*α radiation
μ = 1.59 mm^−1^
*T* = 273 (2) K
0.38 × 0.25 × 0.19 mm

#### Data collection

Bruker SMART CCD area detector diffractometer
Absorption correction: multi-scan (*SADABS*; Sheldrick, 1996 ▶) *T*_min_ = 0.583, *T*_max_ = 0.752
4610 measured reflections
3057 independent reflections
2837 reflections with *I* > 2σ(*I*)
*R*_int_ = 0.015

#### Refinement

*R*[*F* ^2^ > 2σ(*F* ^2^)] = 0.026
*wR*(*F* ^2^) = 0.074
*S* = 1.00
3057 reflections
235 parameters
3 restraints
H-atom parameters constrained
Δρ_max_ = 0.30 e Å^−3^
Δρ_min_ = −0.29 e Å^−3^

### 

Data collection: *SMART* (Siemens, 1996 ▶); cell refinement: *SAINT* (Siemens, 1996 ▶); data reduction: *SAINT*; program(s) used to solve structure: *SHELXS97* (Sheldrick, 2008 ▶); program(s) used to refine structure: *SHELXL97* (Sheldrick, 2008 ▶); molecular graphics: *SHELXTL* (Sheldrick, 1997 ▶); software used to prepare material for publication: *SHELXTL*.

## Supplementary Material

Crystal structure: contains datablocks I, global. DOI: 10.1107/S1600536808000044/cv2370sup1.cif

Structure factors: contains datablocks I. DOI: 10.1107/S1600536808000044/cv2370Isup2.hkl

Additional supplementary materials:  crystallographic information; 3D view; checkCIF report

## Figures and Tables

**Table 1:** Hydrogen-bond geometry (Å, °)

*D*—H⋯*A*	*D*—H	H⋯*A*	*D*⋯*A*	*D*—H⋯*A*
O5—H15⋯O4^i^	0.85	1.96	2.796 (2)	169

Symmetry code: (i) 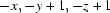.

**Fig. 1.**
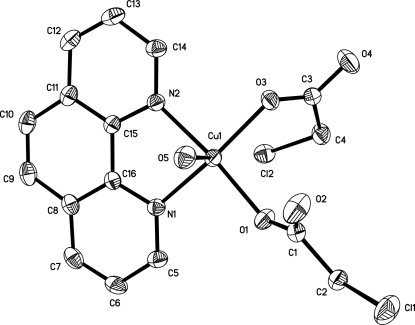
The molecular structure of (I) with atomic numbering and 30% probability displacement ellipsoids.

## supplementary crystallographic information

### Comment

2-Chloroacetic acid and its derivatives are often used in the synthesis of mononuclear monomeric (Sieroń, 2007; Czylkowska *et al.*, 2004) and polymeric compounds (Chen *et al.*, 1996; Overgaard *et al.*, 2003). In our search for new topogical structures, we selected the copper(II) ion with 2-chloroacetic acid in the presence of 1,10-phenanthroline as a co-ligand, and obtained the title compound, (I).

In (I) (Fig. 1), Cu1 exhibits a five-coordinated square-pyramidal environment, formed by two O atoms from two carboxyl ligands (Cu1—O1 1.943 (2) Å, Cu1—O3 1.966 (2) Å), one water molecule (Cu1—O5 2.243 (5) Å) and two N atoms (Cu1—N1 2.029 (2) Å, Cu1—N2 2.005 (2) Å) from 1,10-phenanthroline ligand.

In the crystal structure, there exist short intermolecular Cl···Cl contacts (Table 1), π···π stacking interactions between the aromatic rings from neighbouring molecules (Table 1), and intermolecular O—H···O hydrogen bonds (Table 2), which link the molecules into centrosymmetric dimers.

### Experimental

The reaction was carried out by the solvothermal method. 2-Chloroacetic acid (0.188 g, 2 mmol) and cupric acetate (0.199 g, 1 mmol) and 1,10-phenanthroline (0.180 g, 1 mmol) were added to the airtight vessel with 20 ml water. The resulting green solution was filtered. The filtrate was placed for sevaral days yielding blue block-shaped crystals.

The yield is 81% and elemental analysis: calc. for C_16_H_14_Cl_2_CuN_2_O_5_: C 42.82, H 3.14, N 6.24; found: C 42.55, H 3.39, N 6.32. The elemental analyses were performed with PERKIN ELMER MODEL 2400 SERIES II.

### Refinement

All H atoms were found in Fourier difference map, but placed in idealized positions (C—H 0.93–0.97 Å, O—H 0.85 Å), with *U*_iso_(H)=1.2*U*_eq_ of the parent atom.

### Crystal data

**Table:**

[Cu(C_2_H_2_ClO_2_)_2_(C_12_H_8_N_2_)(H_2_O)]	*Z* = 2
*M**_r_* = 448.73	*F*_000_ = 454
Triclinic, *P*1	*D*_x_ = 1.714 Mg m^−^^3^
*a* = 8.7730 (6) Å	Mo *K*α radiation λ = 0.71073 Å
*b* = 9.2382 (7) Å	Cell parameters from 3430 reflections
*c* = 11.4492 (8) Å	θ = 2.5–28.2º
α = 96.2180 (10)º	µ = 1.59 mm^−^^1^
β = 106.6760 (10)º	*T* = 273 (2) K
γ = 97.9190 (10)º	Block, blue
*V* = 869.66 (11) Å^3^	0.38 × 0.25 × 0.19 mm

### Data collection

**Table:**

Bruker SMART CCD area detector diffractometer	3057 independent reflections
Radiation source: fine-focus sealed tube	2837 reflections with *I* > 2σ(*I*)
Monochromator: graphite	*R*_int_ = 0.015
*T* = 273(2) K	θ_max_ = 25.1º
phi and ω scans	θ_min_ = 1.9º
Absorption correction: multi-scan(SADABS; Sheldrick, 1996)	*h* = −10→10
*T*_min_ = 0.583, *T*_max_ = 0.752	*k* = −8→10
4610 measured reflections	*l* = −13→13

### Refinement

**Table:**

Refinement on *F*^2^	Secondary atom site location: difference Fourier map
Least-squares matrix: full	Hydrogen site location: inferred from neighbouring sites
*R*[*F*^2^ > 2σ(*F*^2^)] = 0.026	H-atom parameters constrained
*wR*(*F*^2^) = 0.074	*w* = 1/[σ^2^(*F*_o_^2^) + (0.043*P*)^2^ + 0.4807*P*] where *P* = (*F*_o_^2^ + 2*F*_c_^2^)/3
*S* = 1.00	(Δ/σ)_max_ = 0.001
3057 reflections	Δρ_max_ = 0.30 e Å^−^^3^
235 parameters	Δρ_min_ = −0.29 e Å^−^^3^
3 restraints	Extinction correction: none
Primary atom site location: structure-invariant direct methods	

### Special details

**Table:**

Geometry. All e.s.d.'s (except the e.s.d. in the dihedral angle between two l.s. planes) are estimated using the full covariance matrix. The cell e.s.d.'s are taken into account individually in the estimation of e.s.d.'s in distances, angles and torsion angles; correlations between e.s.d.'s in cell parameters are only used when they are defined by crystal symmetry. An approximate (isotropic) treatment of cell e.s.d.'s is used for estimating e.s.d.'s involving l.s. planes.
Refinement. Refinement of *F*^2^ against ALL reflections. The weighted *R*-factor *wR* and goodness of fit *S* are based on *F*^2^, conventional *R*-factors *R* are based on *F*, with *F* set to zero for negative *F*^2^. The threshold expression of *F*^2^ > σ(*F*^2^) is used only for calculating *R*-factors(gt) *etc*. and is not relevant to the choice of reflections for refinement. *R*-factors based on *F*^2^ are statistically about twice as large as those based on *F*, and *R*- factors based on ALL data will be even larger.

### Fractional atomic coordinates and isotropic or equivalent isotropic displacement parameters (Å^2^)

**Table:**

	*x*	*y*	*z*	*U*_iso_*/*U*_eq_	
Cu1	0.23816 (3)	0.68495 (3)	0.71095 (2)	0.03304 (10)	
Cl1	−0.22927 (8)	0.98116 (9)	0.83337 (8)	0.0671 (2)	
Cl2	0.40479 (7)	0.93989 (7)	0.62804 (6)	0.04661 (16)	
O1	0.14841 (19)	0.84228 (17)	0.77963 (15)	0.0421 (4)	
O2	−0.1084 (2)	0.7271 (2)	0.7366 (2)	0.0635 (5)	
O3	0.11593 (19)	0.68927 (16)	0.53849 (14)	0.0408 (4)	
O4	0.0053 (2)	0.7855 (2)	0.37496 (17)	0.0622 (5)	
O5	0.06637 (19)	0.50494 (17)	0.74928 (15)	0.0430 (4)	
H15	0.0312	0.4180	0.7091	0.052*	
H16	−0.0123	0.5517	0.7385	0.052*	
N1	0.4251 (2)	0.70913 (19)	0.86886 (16)	0.0328 (4)	
N2	0.3550 (2)	0.53104 (19)	0.65745 (16)	0.0334 (4)	
C1	0.0003 (3)	0.8353 (2)	0.77567 (19)	0.0365 (5)	
C2	−0.0300 (3)	0.9841 (3)	0.8242 (2)	0.0400 (5)	
H2A	−0.0099	1.0543	0.7708	0.048*	
H2B	0.0464	1.0189	0.9057	0.048*	
C3	0.1023 (3)	0.7947 (2)	0.4784 (2)	0.0366 (5)	
C4	0.2075 (3)	0.9464 (3)	0.5315 (2)	0.0488 (6)	
H4A	0.1539	1.0031	0.5788	0.059*	
H4B	0.2171	0.9981	0.4639	0.059*	
C5	0.4552 (3)	0.7987 (3)	0.9743 (2)	0.0404 (5)	
H5A	0.3851	0.8640	0.9805	0.049*	
C6	0.5887 (3)	0.7988 (3)	1.0767 (2)	0.0486 (6)	
H6	0.6071	0.8642	1.1490	0.058*	
C7	0.6915 (3)	0.7028 (3)	1.0699 (2)	0.0469 (6)	
H7	0.7797	0.7014	1.1380	0.056*	
C8	0.6639 (3)	0.6057 (3)	0.9596 (2)	0.0388 (5)	
C9	0.7622 (3)	0.4983 (3)	0.9421 (2)	0.0479 (6)	
H9	0.8521	0.4911	1.0067	0.057*	
C10	0.7268 (3)	0.4079 (3)	0.8339 (3)	0.0477 (6)	
H10	0.7934	0.3401	0.8249	0.057*	
C11	0.5883 (3)	0.4140 (2)	0.7322 (2)	0.0387 (5)	
C12	0.5395 (3)	0.3200 (3)	0.6177 (2)	0.0453 (6)	
H12	0.6011	0.2500	0.6028	0.054*	
C13	0.4019 (3)	0.3317 (3)	0.5290 (2)	0.0460 (6)	
H13	0.3688	0.2690	0.4537	0.055*	
C14	0.3111 (3)	0.4378 (2)	0.5512 (2)	0.0390 (5)	
H14	0.2168	0.4439	0.4901	0.047*	
C15	0.4909 (2)	0.5181 (2)	0.74641 (19)	0.0319 (4)	
C16	0.5289 (2)	0.6148 (2)	0.86151 (19)	0.0317 (4)	

### Atomic displacement parameters (Å^2^)

**Table:**

	*U*^11^	*U*^22^	*U*^33^	*U*^12^	*U*^13^	*U*^23^
Cu1	0.03296 (16)	0.03095 (16)	0.03298 (16)	0.00803 (11)	0.00661 (11)	0.00203 (10)
Cl1	0.0439 (4)	0.0748 (5)	0.0858 (5)	0.0241 (3)	0.0227 (3)	0.0012 (4)
Cl2	0.0413 (3)	0.0447 (3)	0.0484 (3)	−0.0015 (2)	0.0122 (3)	−0.0006 (3)
O1	0.0360 (8)	0.0378 (8)	0.0516 (9)	0.0094 (7)	0.0135 (7)	−0.0010 (7)
O2	0.0409 (10)	0.0442 (10)	0.0972 (16)	0.0042 (8)	0.0163 (10)	−0.0071 (10)
O3	0.0474 (9)	0.0287 (8)	0.0373 (8)	0.0040 (7)	−0.0002 (7)	0.0058 (6)
O4	0.0747 (13)	0.0462 (10)	0.0453 (10)	0.0019 (9)	−0.0113 (9)	0.0129 (8)
O5	0.0435 (9)	0.0342 (8)	0.0493 (9)	0.0040 (7)	0.0130 (7)	0.0051 (7)
N1	0.0344 (9)	0.0302 (9)	0.0328 (9)	0.0031 (7)	0.0105 (7)	0.0032 (7)
N2	0.0359 (9)	0.0312 (9)	0.0328 (9)	0.0052 (7)	0.0109 (8)	0.0037 (7)
C1	0.0357 (12)	0.0380 (12)	0.0352 (11)	0.0099 (10)	0.0078 (9)	0.0069 (9)
C2	0.0376 (12)	0.0416 (12)	0.0415 (12)	0.0122 (10)	0.0117 (10)	0.0041 (10)
C3	0.0399 (12)	0.0330 (11)	0.0340 (11)	0.0085 (9)	0.0069 (9)	0.0025 (9)
C4	0.0540 (15)	0.0334 (12)	0.0501 (14)	0.0061 (11)	0.0024 (12)	0.0077 (10)
C5	0.0457 (13)	0.0362 (12)	0.0372 (12)	0.0049 (10)	0.0129 (10)	−0.0011 (9)
C6	0.0548 (15)	0.0482 (14)	0.0327 (12)	−0.0027 (12)	0.0063 (11)	−0.0022 (10)
C7	0.0418 (13)	0.0490 (14)	0.0390 (12)	−0.0038 (11)	−0.0005 (10)	0.0114 (11)
C8	0.0344 (11)	0.0398 (12)	0.0409 (12)	0.0010 (9)	0.0090 (9)	0.0142 (10)
C9	0.0337 (12)	0.0546 (15)	0.0570 (15)	0.0110 (11)	0.0099 (11)	0.0230 (12)
C10	0.0400 (13)	0.0467 (14)	0.0657 (16)	0.0188 (11)	0.0217 (12)	0.0192 (12)
C11	0.0396 (12)	0.0326 (11)	0.0516 (13)	0.0075 (9)	0.0231 (10)	0.0129 (10)
C12	0.0530 (15)	0.0319 (12)	0.0609 (15)	0.0109 (10)	0.0316 (13)	0.0060 (10)
C13	0.0582 (15)	0.0329 (12)	0.0474 (14)	0.0002 (11)	0.0247 (12)	−0.0048 (10)
C14	0.0436 (12)	0.0339 (11)	0.0363 (11)	0.0008 (9)	0.0121 (10)	0.0005 (9)
C15	0.0326 (11)	0.0288 (10)	0.0370 (11)	0.0040 (8)	0.0145 (9)	0.0080 (8)
C16	0.0302 (10)	0.0313 (10)	0.0341 (11)	0.0024 (8)	0.0107 (9)	0.0091 (8)

### Geometric parameters (Å, °)

**Table:**

Cu1—O1	1.9427 (15)	C4—H4A	0.9700
Cu1—O3	1.9657 (15)	C4—H4B	0.9700
Cu1—N2	2.0052 (18)	C5—C6	1.399 (3)
Cu1—N1	2.0294 (18)	C5—H5A	0.9300
Cu1—O5	2.2531 (16)	C6—C7	1.361 (4)
Cl1—C2	1.778 (2)	C6—H6	0.9300
Cl1—Cl2^i^	3.3340 (10)	C7—C8	1.406 (3)
Cl2—C4	1.779 (3)	C7—H7	0.9300
O1—C1	1.279 (3)	C8—C16	1.398 (3)
O2—C1	1.226 (3)	C8—C9	1.435 (3)
O3—C3	1.251 (3)	C9—C10	1.346 (4)
O4—C3	1.231 (3)	C9—H9	0.9300
O5—H15	0.8498	C10—C11	1.434 (3)
O5—H16	0.8498	C10—H10	0.9300
N1—C5	1.324 (3)	C11—C15	1.398 (3)
N1—C16	1.357 (3)	C11—C12	1.408 (3)
N2—C14	1.336 (3)	C12—C13	1.363 (4)
N2—C15	1.357 (3)	C12—H12	0.9300
C1—C2	1.514 (3)	C13—C14	1.392 (3)
C2—H2A	0.9700	C13—H13	0.9300
C2—H2B	0.9700	C14—H14	0.9300
C3—C4	1.524 (3)	C15—C16	1.434 (3)
			
Cl1···Cl2^i^	3.334 (1)	Cg1···Cg2^ii^	3.621 (11)
			
O1—Cu1—O3	94.93 (7)	H4A—C4—H4B	107.7
O1—Cu1—N2	173.08 (7)	N1—C5—C6	122.4 (2)
O3—Cu1—N2	91.04 (7)	N1—C5—H5A	118.8
O1—Cu1—N1	91.67 (7)	C6—C5—H5A	118.8
O3—Cu1—N1	160.92 (7)	C7—C6—C5	119.7 (2)
N2—Cu1—N1	81.58 (7)	C7—C6—H6	120.2
O1—Cu1—O5	93.30 (6)	C5—C6—H6	120.2
O3—Cu1—O5	98.18 (6)	C6—C7—C8	119.7 (2)
N2—Cu1—O5	89.32 (7)	C6—C7—H7	120.1
N1—Cu1—O5	99.28 (7)	C8—C7—H7	120.1
C1—O1—Cu1	125.47 (14)	C16—C8—C7	116.6 (2)
C3—O3—Cu1	130.63 (14)	C16—C8—C9	118.6 (2)
Cu1—O5—H15	127.2	C7—C8—C9	124.8 (2)
Cu1—O5—H16	95.7	C10—C9—C8	121.3 (2)
H15—O5—H16	108.2	C10—C9—H9	119.3
C5—N1—C16	117.97 (19)	C8—C9—H9	119.3
C5—N1—Cu1	129.49 (16)	C9—C10—C11	121.2 (2)
C16—N1—Cu1	112.52 (13)	C9—C10—H10	119.4
C14—N2—C15	118.05 (19)	C11—C10—H10	119.4
C14—N2—Cu1	128.50 (16)	C15—C11—C12	116.5 (2)
C15—N2—Cu1	113.32 (13)	C15—C11—C10	118.7 (2)
O2—C1—O1	127.3 (2)	C12—C11—C10	124.8 (2)
O2—C1—C2	121.8 (2)	C13—C12—C11	119.9 (2)
O1—C1—C2	110.88 (19)	C13—C12—H12	120.0
C1—C2—Cl1	113.88 (16)	C11—C12—H12	120.0
C1—C2—H2A	108.8	C12—C13—C14	119.9 (2)
Cl1—C2—H2A	108.8	C12—C13—H13	120.0
C1—C2—H2B	108.8	C14—C13—H13	120.0
Cl1—C2—H2B	108.8	N2—C14—C13	122.0 (2)
H2A—C2—H2B	107.7	N2—C14—H14	119.0
O4—C3—O3	123.8 (2)	C13—C14—H14	119.0
O4—C3—C4	115.3 (2)	N2—C15—C11	123.6 (2)
O3—C3—C4	120.9 (2)	N2—C15—C16	116.28 (18)
C3—C4—Cl2	113.92 (17)	C11—C15—C16	120.1 (2)
C3—C4—H4A	108.8	N1—C16—C8	123.6 (2)
Cl2—C4—H4A	108.8	N1—C16—C15	116.28 (18)
C3—C4—H4B	108.8	C8—C16—C15	120.1 (2)
Cl2—C4—H4B	108.8		
			
O3—Cu1—O1—C1	65.71 (18)	C5—C6—C7—C8	0.8 (4)
N2—Cu1—O1—C1	−144.9 (5)	C6—C7—C8—C16	0.0 (3)
N1—Cu1—O1—C1	−132.21 (18)	C6—C7—C8—C9	−179.0 (2)
O5—Cu1—O1—C1	−32.81 (18)	C16—C8—C9—C10	0.2 (3)
O1—Cu1—O3—C3	52.0 (2)	C7—C8—C9—C10	179.2 (2)
N2—Cu1—O3—C3	−124.4 (2)	C8—C9—C10—C11	−0.7 (4)
N1—Cu1—O3—C3	−57.7 (3)	C9—C10—C11—C15	1.0 (3)
O5—Cu1—O3—C3	146.1 (2)	C9—C10—C11—C12	−177.6 (2)
O1—Cu1—N1—C5	2.6 (2)	C15—C11—C12—C13	−1.2 (3)
O3—Cu1—N1—C5	112.9 (2)	C10—C11—C12—C13	177.4 (2)
N2—Cu1—N1—C5	−178.9 (2)	C11—C12—C13—C14	0.7 (4)
O5—Cu1—N1—C5	−91.01 (19)	C15—N2—C14—C13	−1.5 (3)
O1—Cu1—N1—C16	−179.12 (14)	Cu1—N2—C14—C13	−177.15 (17)
O3—Cu1—N1—C16	−68.8 (3)	C12—C13—C14—N2	0.7 (4)
N2—Cu1—N1—C16	−0.65 (14)	C14—N2—C15—C11	0.9 (3)
O5—Cu1—N1—C16	87.26 (14)	Cu1—N2—C15—C11	177.24 (16)
O1—Cu1—N2—C14	−170.8 (5)	C14—N2—C15—C16	−176.64 (18)
O3—Cu1—N2—C14	−21.29 (19)	Cu1—N2—C15—C16	−0.3 (2)
N1—Cu1—N2—C14	176.37 (19)	C12—C11—C15—N2	0.4 (3)
O5—Cu1—N2—C14	76.88 (19)	C10—C11—C15—N2	−178.3 (2)
O1—Cu1—N2—C15	13.4 (6)	C12—C11—C15—C16	177.90 (19)
O3—Cu1—N2—C15	162.87 (14)	C10—C11—C15—C16	−0.8 (3)
N1—Cu1—N2—C15	0.53 (14)	C5—N1—C16—C8	1.0 (3)
O5—Cu1—N2—C15	−98.96 (14)	Cu1—N1—C16—C8	−177.50 (16)
Cu1—O1—C1—O2	5.6 (3)	C5—N1—C16—C15	179.16 (18)
Cu1—O1—C1—C2	−172.99 (14)	Cu1—N1—C16—C15	0.7 (2)
O2—C1—C2—Cl1	6.1 (3)	C7—C8—C16—N1	−1.0 (3)
O1—C1—C2—Cl1	−175.31 (16)	C9—C8—C16—N1	178.1 (2)
Cu1—O3—C3—O4	−170.39 (19)	C7—C8—C16—C15	−179.08 (19)
Cu1—O3—C3—C4	9.1 (3)	C9—C8—C16—C15	0.0 (3)
O4—C3—C4—Cl2	−147.4 (2)	N2—C15—C16—N1	−0.2 (3)
O3—C3—C4—Cl2	33.1 (3)	C11—C15—C16—N1	−177.89 (18)
C16—N1—C5—C6	−0.1 (3)	N2—C15—C16—C8	178.01 (18)
Cu1—N1—C5—C6	178.14 (17)	C11—C15—C16—C8	0.3 (3)
N1—C5—C6—C7	−0.8 (4)		

Symmetry codes: (i) *x*−1, *y*, *z*; (ii) −*x*+1, −*y*+1, −*z*+2.

### Hydrogen-bond geometry (Å, °)

**Table:**

*D*—H···*A*	*D*—H	H···*A*	*D*···*A*	*D*—H···*A*
O5—H15···O4^iii^	0.85	1.96	2.796 (2)	169

Symmetry codes: (iii) −*x*, −*y*+1, −*z*+1.
